# Impaired Treg-DC interactions contribute to autoimmunity in leukocyte adhesion deficiency type 1

**DOI:** 10.1172/jci.insight.162580

**Published:** 2022-12-22

**Authors:** Tanja Klaus, Alicia S. Wilson, Elisabeth Vicari, Eva Hadaschik, Matthias Klein, Sara Salome Clara Helbich, Nadine Kamenjarin, Katrin Hodapp, Jenny Schunke, Maximilian Haist, Florian Butsch, Hans Christian Probst, Alexander H. Enk, Karsten Mahnke, Ari Waisman, Monika Bednarczyk, Matthias Bros, Tobias Bopp, Stephan Grabbe

**Affiliations:** 1 Department of Dermatology,; 2 Research Center for Immunotherapy, and; 3 Institute of Immunology, University of Mainz Medical Center, Mainz, Germany.; 4 Department of Dermatology, University of Heidelberg, Heidelberg, Germany.; 5 Department of Dermatology, University Hospital Essen, Essen, Germany.; 6 Institute for Molecular Medicine, University of Mainz Medical Center, Mainz, Germany.

**Keywords:** Autoimmunity, Autoimmune diseases, T cells

## Abstract

Leukocyte adhesion deficiency type 1 (LAD-1) is a rare disease resulting from mutations in the gene encoding for the common β-chain of the β_2_-integrin family (CD18). The most prominent clinical symptoms are profound leukocytosis and high susceptibility to infections. Patients with LAD-1 are prone to develop autoimmune diseases, but the molecular and cellular mechanisms that result in coexisting immunodeficiency and autoimmunity are still unresolved. CD4^+^FOXP3^+^ Treg are known for their essential role in preventing autoimmunity. To understand the role of Treg in LAD-1 development and manifestation of autoimmunity, we generated mice specifically lacking CD18 on Treg (CD18^Foxp3^), resulting in defective LFA-1 expression. Here, we demonstrate a crucial role of LFA-1 on Treg to maintain immune homeostasis by modifying T cell–DC interactions and CD4^+^ T cell activation. Treg-specific CD18 deletion did not impair Treg migration into extralymphatic organs, but it resulted in shorter interactions of Treg with DC. In vivo, CD18^Foxp3^ mice developed spontaneous hyperplasia in lymphatic organs and diffuse inflammation of the skin and in multiple internal organs. Thus, LFA-1 on Treg is required for the maintenance of immune homeostasis.

## Graphical abstract

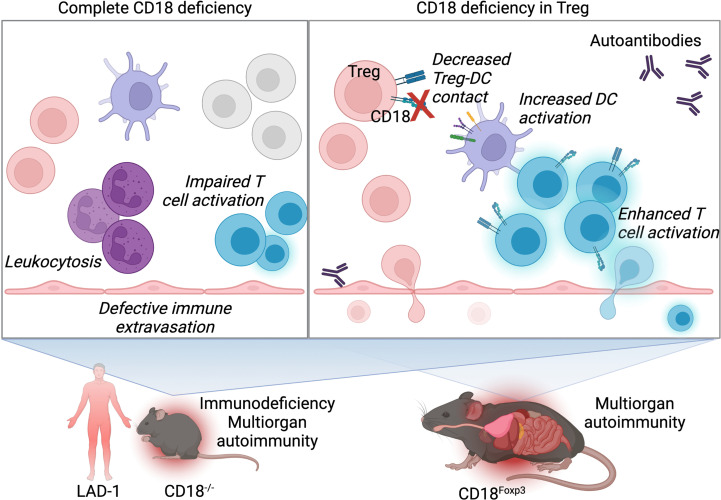

## Introduction

β_2_-Integrins are heterodimeric surface receptors that play important roles in cell-to-cell communication and in the extravasation of leukocytes from blood into inflamed tissues (1, 2). They consist of a variable α-chain (CD11a, CD11b, CD11c, CD11d) and a constant β-chain (CD18), which are expressed in a cell-specific pattern by all leukocytes (3). Lymphocyte function–associated antigen 1 (LFA-1; CD11a/CD18), the only β_2_-integrin expressed on T cells, orchestrates the immunological synapse (IS) by binding to intercellular cell adhesion molecules (ICAMs) on antigen-presenting cells (APC). In response to inside-out signaling, LFA-1 switches from an inactive low- to a high-affinity state, allowing long-lasting T cell–APC interactions, which are essential for the full activation of T cells and effective immune responses (4). CD4^+^FOXP3^+^ Treg are crucial for the maintenance of peripheral tolerance and the regulation of immune responses. The immunosuppressive function of Treg depends on different mechanisms and soluble factors, including cell migration to sites of inflammation, cytokine production, and suppressive interactions with APC (5–8). Mutational inactivation of FOXP3 — which causes the loss of functional Treg, such as in IPEX syndrome — leads to severe multiorgan autoimmunity (9, 10).

In humans, dysfunctional CD18 leads to a rare disease known as leukocyte adhesion deficiency type 1 (LAD-1), in which the CD18 protein is not present or mutated and is, therefore, unable to form correct β_2_-integrin complexes with the α subunit. Consequently, all β_2_-integrins display 20%–30% residual expression or are even completely absent. Most patients with a severe LAD-1 phenotype completely lacking β_2_-integrins die in early childhood (11). In general, patients with LAD-1 suffer from persistent leukocytosis, recurrent infections due to defective leukocyte adhesion, extravasation, wound-healing defects, and autoimmune-mediated symptoms (12–14). So far, several mouse models have been established to investigate the role of β_2_-integrins through global deletion of either the α or β subunit (15, 16). In contrast to CD11a^–/–^ mice with no significant phenotype (17), CD18 hypomorphic (CD18^hypo^) PL/J mice with up to 16% residual expression of CD18 are characterized by a skin disease that closely resembles human psoriasis (18, 19). Complete deficiency of CD18 (CD18^–/–^) results in a phenotype closely similar to patients with LAD-1 with a lack of lymphocyte and granulocyte egress into tissues (15, 20, 21). These CD18^–/–^ mice also display spontaneous skin inflammation, dysregulation of lymphatic organs, and defective T cell function (15, 21). These strains, in which the β subunit of LFA-1 was constitutively deleted, recapitulate the leukocytosis, immune deficiency, and seemingly contradictory autoimmune aspects of LAD-1. However, since all leukocytes are affected, it is difficult to distinguish the direct contribution of specific cells to the various phenotypic alterations (3). Thus, to analyze the exact relevance of β_2_-integrins for cell-specific contributions to LAD-1 pathology, we generated a mouse model with floxed CD18 (CD18^fl/fl^) alleles that were crossed with Foxp3^cre^-expressing mice, resulting in mice with a cell-specific knockdown of LFA-1 (CD18^Foxp3^) only on Treg. These mice spontaneously develop dermatitis, enlarged lymphatic organs, and systemic multiorgan immune activation.

In this report, we characterized skin and peripheral organ inflammation regarding immune cell distributions and activation states in CD18^Foxp3^ mice. These mice have an increased frequency of activated CD4 conventional T cells (Tconv) in multiple organs that is not due to a defect in CD18-deficient Treg homing. Treg from CD18^Foxp3^ mice were found to be distributed normally through tissues and even expanded in some sites, despite lack of LFA-1–mediated migration. Furthermore, LFA-1 deficiency on Treg resulted in impaired cell-to-cell contacts and reduced contact time with DC, followed by increased activation of DC in CD18^Foxp3^ mice. To our knowledge, these CD18^Foxp3^ mice provide the first murine model to investigate the role of LFA-1 on Treg for immune regulation. Our data suggest that expression of LFA-1 by Treg is indispensable to maintain immune homeostasis and implicate that CD18-deficient Treg are critical for the autoimmune pathology seen in LAD-1 patients.

## Results

### The specific deletion of β_2_-integrins in Treg leads to the spontaneous development of skin inflammation.

**Figure 1:**
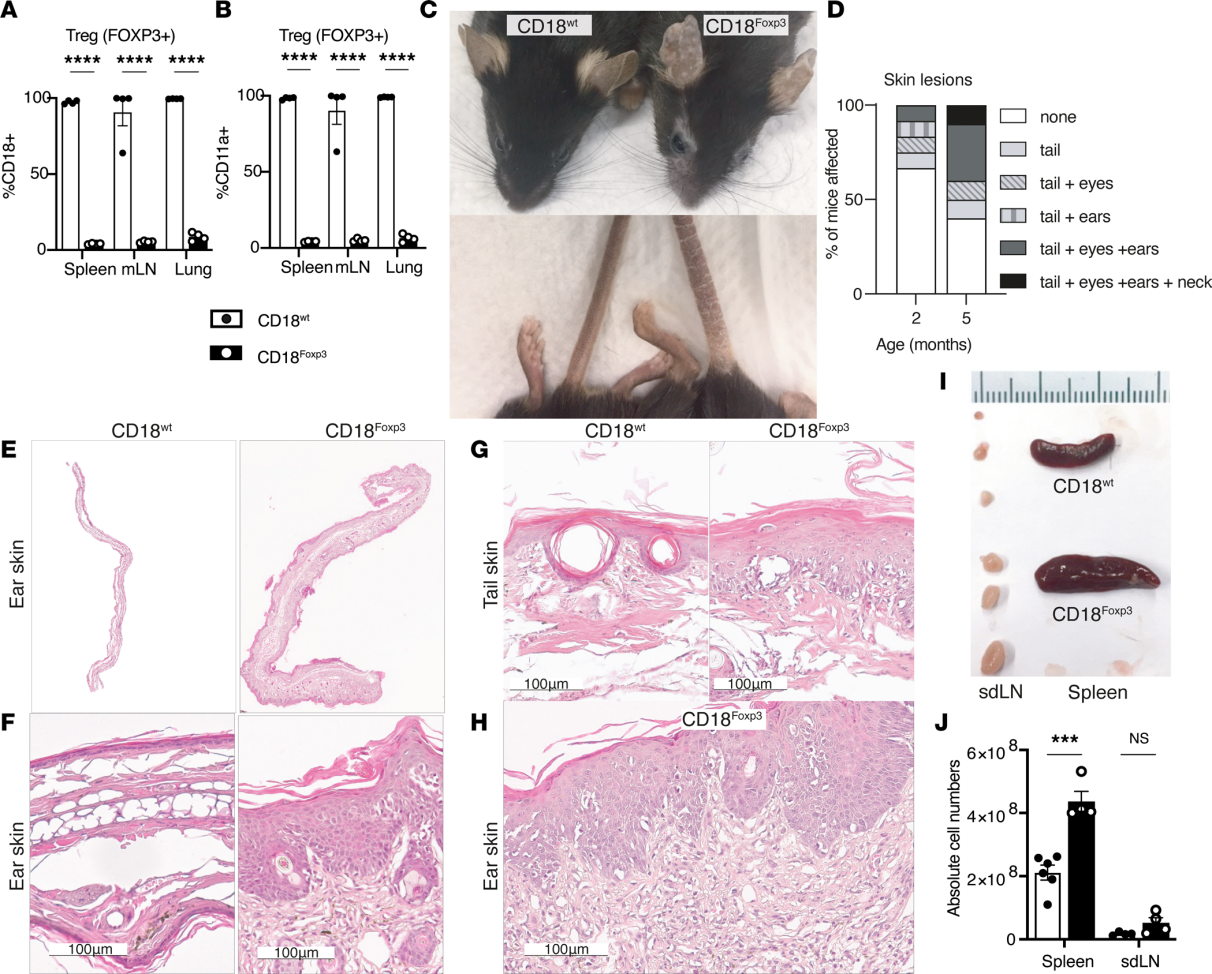
Treg-specific deletion of CD18 leads to the development of spontaneous skin inflammation. (**A** and **B**) CD18 and CD11a expression in (FOXP3^+^) Treg measured by flow cytometry. *n* = 4. (**C**) Representative images of spontaneous skin inflammation developed by CD18^Foxp3^ mice at 20 weeks of age. (**D**) Disease progression of skin lesions developed by CD18^Foxp3^ mice between 2 and 5 months of age (10–12 female and male mice were observed). (**E**–**H**) Representative images of H&E staining of ear and tail skin tissue of 21- to 22-week old mice at 10× (**E**), 20× (ear) (**F**), 20× (tail) (**G**), and 200× (**H**) magnification. (**I**) Representative pictures of spontaneous hyperplasia of spleen and skin-draining lymph nodes (sdLN) of 12-week-old CD18^Foxp3^ mice compared with littermate controls. Scale indicates 1 mm increments. (**J**) Absolute cell numbers from spleens and sdLN of 12-week-old mice. *n* = 4–6. Dots represent individual mice. Data are shown as mean ± SEM. Significance determined by 2-way ANOVA with Šídák’s multiple-comparison test (**A** and **B**) or multiple unpaired *t* tests corrected for multiple comparisons by the 2-stage step-up (Benjamini, Krieger, and Yekutieli) method (**J**). ***P ≤ 0.001; ****P ≤ 0.0001.

Alongside persistent leukocytosis and recurrent infections, patients with LAD-1, lacking functional CD18, develop autoimmune syndromes (12–14). While it has been postulated that Treg from patients with secondary mutations restoring CD18 function in some cells (reversion mutations) display reduced suppressive activity and may contribute to the development of intestinal bowel disease (IBD) in these patients (14), their direct contribution to the induction of autoimmunity remains unclear. To directly address the role of Treg-expressed CD18, we generated a mouse strain lacking CD18 expression in FOXP3-expressing cells (Supplemental Figures 1 and 2; supplemental material available online with this article; https://doi.org/10.1172/jci.insight.162580DS1). Deletion of CD18 in FOXP3^+^ cells was confirmed by flow cytometry in several organs (Figure 1A). The export of functional integrins from the endoplasmic reticulum is reliant of the expression of both α and β subunits of the heterodimer. In the absence of β subunit expression, α subunits are not stabilized and are readily degraded (22). Thus, to further verify the functional deletion of CD18, the concomitant loss of the surface expression the β_2_-integrin LFA-1 α chain CD11a was confirmed (Figure 1B). Expression of CD18 and CD11a remained at WT levels in FOXP3^–^CD4^+^ Tconv (Supplemental Figure 3A) and in CD8^+^ T cells (Supplemental Figure 3B), confirming the Treg-specific loss of CD18.

CD18^Foxp3^ mice housed in specific pathogen–free conditions spontaneously developed skin inflammation characterized by scaling and thickening of distinct regions (Figure 1C). Skin lesions were most commonly observed on the tails and faces of mice, with the number of sites of affected skin increasing with age (Figure 1D). Histological analysis of inflamed ears showed thickening of the dermis and epidermis of CD18^Foxp3^ mice (Figure 1E); gross increases in ear thickness were also confirmed (Supplemental Figure 3C). Increased magnification of H&E-stained ear sections revealed severe epidermal acanthosis, parakeratosis, and moderate spongiosis in CD18^Foxp3^ mouse ears and tails (Figure 1, F and G). Further examination of the dermal compartment showed a mixed polymorphonuclear infiltrate of inflammatory cells into the epidermis (Figure 1H). Affected CD18^Foxp3^ mice also developed profound hyperplasia of the spleen and skin-draining lymph nodes (Figure 1I). Quantification of the total number of splenocytes confirmed greatly increased Tconv numbers in the spleens of older mice (Figure 1J), indicative of a systemic immune response.

CD18 can form heterodimers with multiple α-integrins, including CD11a (αLβ_2_, LFA-1), CD11b (αMβ_2_, Mac-1, CR3), CD11c (αXβ_2_, p150.95, CR4), and CD11d (αDβ_2_); the deletion of CD18 could lead to the functional loss of multiple integrins. Since signals mediated by αMβ_2_ can have immune-inhibitory effects in some myeloid cell populations (23), we thus examined which integrin complexes are expressed in Treg and whether these were all equally affected by the loss of β_2_-integrin expression. Analysis of gene expression from sorted Treg showed hardly detectable expression of α-integrins *Itgad*, *Itgam*, and *Itgax* on the mRNA level (Supplemental Figure 3D). *Itgal* mRNA, on the other hand, was highly expressed, with expression slightly decreased in CD18^Foxp3^ mice (Supplemental Figure 3D). The same expression pattern of these α-integrins was also observed in Treg stimulated ex vivo (Supplemental Figure 3E). We confirmed this expression pattern for CD11a, CD11b, and CD11c on the protein level by flow cytometry, where, in line with previously published reports, only CD11a was highly expressed on Treg (Supplemental Figure 3F; ref. 24). Here, too, the loss of CD18 in Treg only affected the expression of CD11a, implicating the loss of LFA-1 (CD11a/CD18) as the main driver of the phenotype observed.

### Absence of CD18 expression on Treg leads to multiorgan leukocyte infiltration.

Treg in peripheral organs play critical roles in regulating immune tolerance and inflammation. A defect in their function leads to a variety of inflammatory as well as autoimmune disorders that manifest not only in the skin, but also occur as multiorgan dysregulations (25). The transcription factor FOXP3 is known to be essential for their correct regulatory function. Scurfy mice with a mutation in FOXP3 exhibit a defect in their suppressive capacity, resulting in multiple autoimmune phenomena resembling human immunodysregulation, polyendocrinopathy, enteropathy, X-linked (IPEX) syndrome (26, 27).

**Figure 2:**
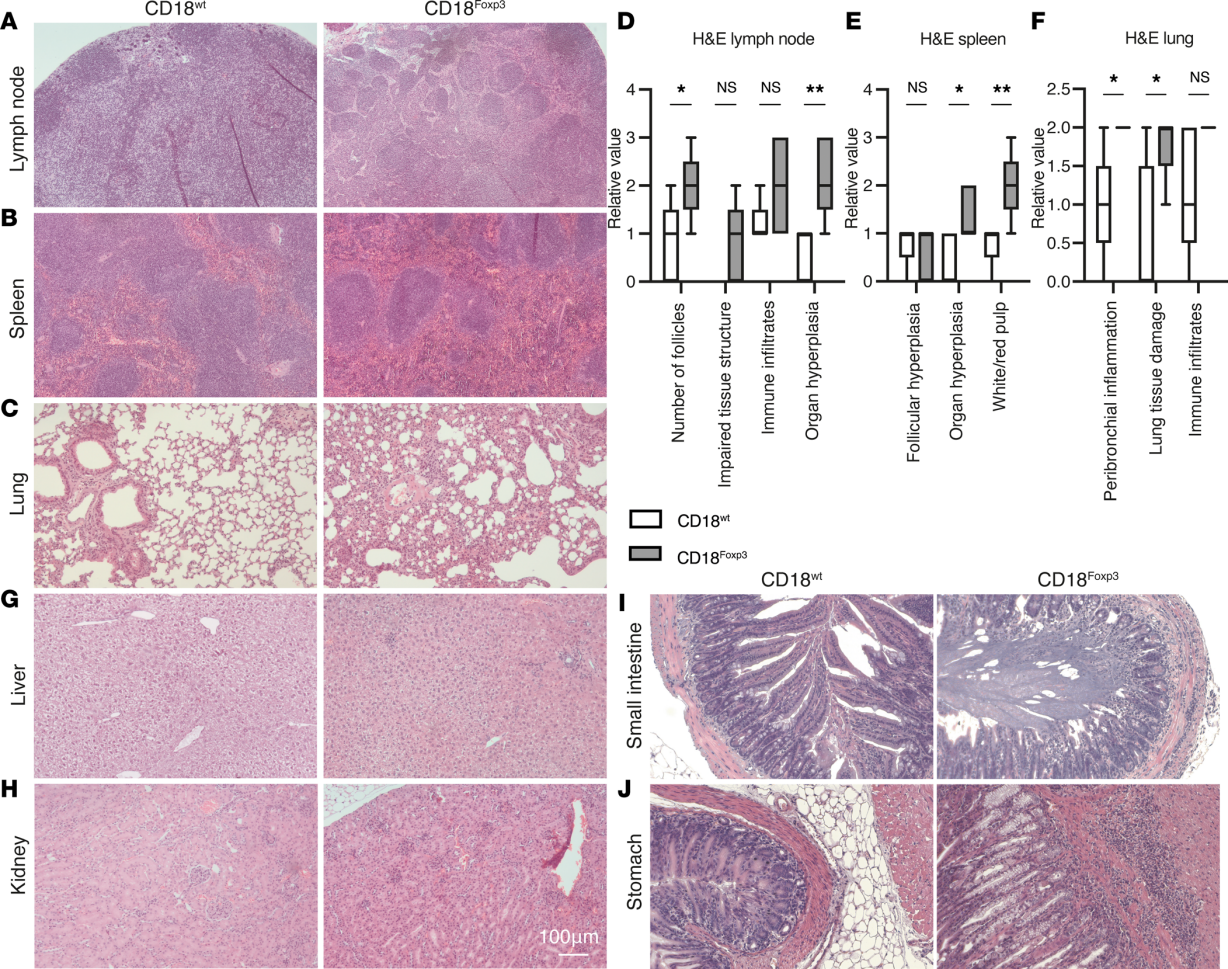
Treg-specific deletion of CD18 leads to multiorgan autoimmunity. (**A**–**C**) Representative images of H&E-stained cross sections of the lymph node, spleen, and lungs of 11- to 15-week-old CD18^wt^ and CD18^Foxp3^ mice. (**D**–**F**) Quantification of H&E staining from lymph node, spleen, and lung samples from CD18^wt^ and CD18^Foxp3^ mice *n* = 5. (**G**–**J**) Representative images of H&E stained cross sections of the liver, kidney, small intestine, and stomach of 11- to 15-week-old CD18^wt^ and CD18^Foxp3^ mice. (**D**–**F**) Box plots extend from the 25th to the 75th percentile, and whiskers indicate the 5th and 95th percentiles. Significance was determined by 2-way ANOVA with Šídák’s multiple-comparison test. *P < 0.05; **P < 0.01.

To determine whether the observed skin inflammation in CD18^Foxp3^ mice was a product of a skin-specific inflammatory process or a sign of a systemic break in tolerance, as suggested by the splenic hyperplasia, we examined immune infiltration in various organs. Histopathological examination of H&E-stained sections revealed a significant hyperplasia of lymph nodes from CD18^Foxp3^ mice. Furthermore, there was a substantial increase in tertiary follicular structures within the lymph nodes and a prominent efferent vessel ectasia (Figure 2A). In spleens of CD18^Foxp3^ mice, our observations indicated a relative hypoplasia of primary follicles, whereas the red pulp and parafollicular T cell zone were more prominent (Figure 2B). Lungs of CD18^Foxp3^ mice showed significantly stronger lymphocytic and monocytic infiltration and substantial lung damage, with atelectasis and exudation into lung alveoli (Figure 2C). Quantification of H&E staining from these organs confirmed organ hyperplasia, increased follicle number in lymph nodes, altered white/red pulp ratios, and lung damage in CD18^Foxp3^ mice (Figure 2, D–F).

In the liver, a tendency toward stronger lymphocytic infiltration in the periportal fields of CD18^Foxp3^ mice was also observed (Figure 2G and Supplemental Figure 4A). By contrast, kidneys of both genotypes did not show significant differences in terms of immune cell infiltration, inflammation, or organ injury (Figure 2H), although, in kidneys of CD18^FoxP3^ mice, there was a tendency toward a stronger periglomerular immune cell infiltrate (Supplemental Figure 4B). Histological analysis of cross-sections of the small intestine revealed an inflammatory infiltrate in the lamina muscularis mucosae and the lower part of the mucosa in CD18^Foxp3^ mice (Figure 2I). Cross-sections of the gastric wall revealed an inflammatory infiltrate in the muscular layer, the lamina propria, and the lower part of the gastric mucosa in CD18^Foxp3^ mice (Figure 2J). Overall, immune infiltrates in the gastrointestinal tract (GIT) were more commonly observed in CD18^Foxp3^ mice than in their littermate controls (Supplemental Figure 4C).

### Multiorgan inflammation in mice lacking CD18 on Treg leads to spontaneous development of autoantibodies.

**Figure 3:**
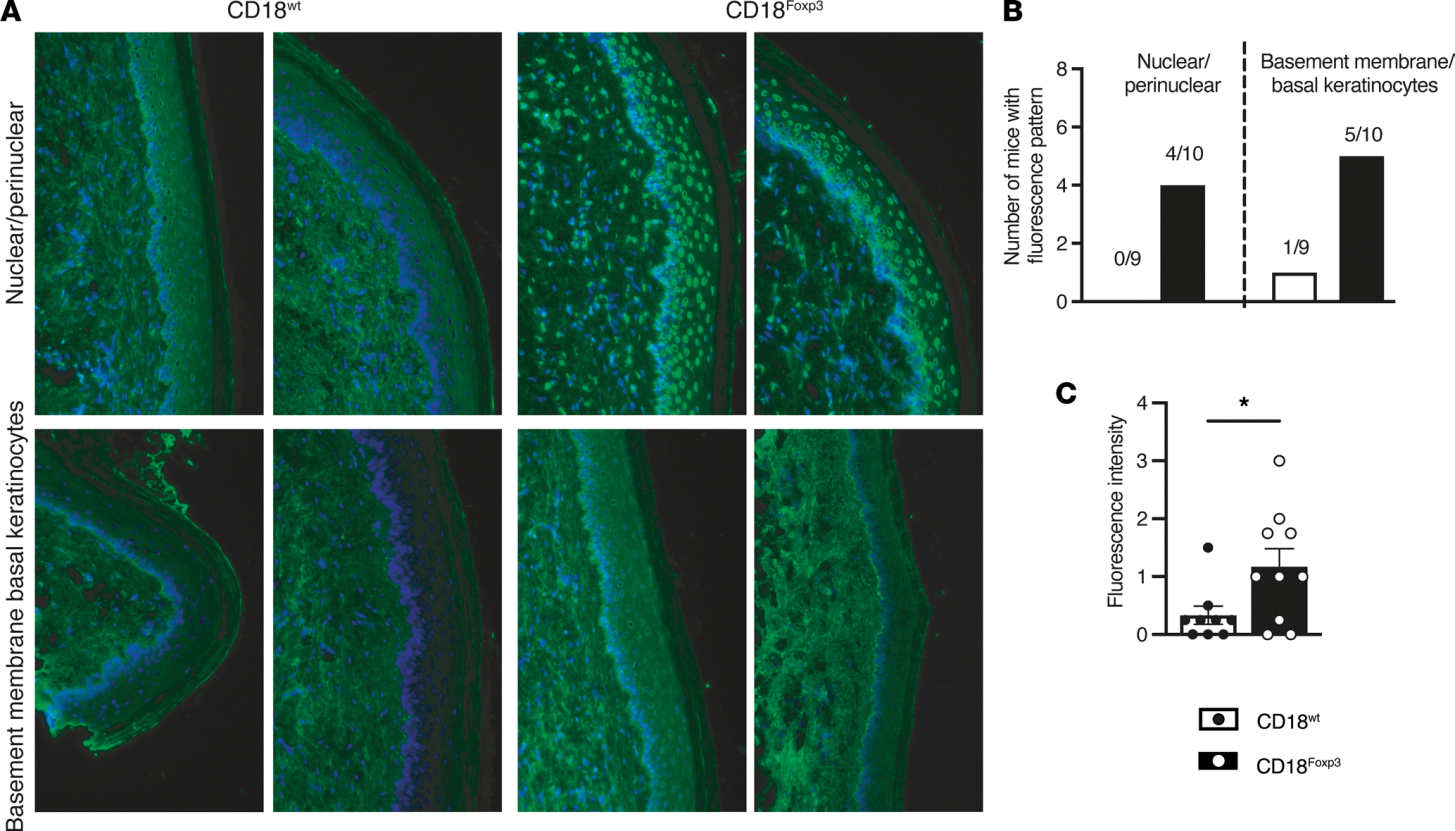
Absence of CD18 expression on Treg leads to the spontaneous generation of autoantibodies. (**A**) Representative images of autoantibody binding patterns of CD18^Foxp3^ and CD18^wt^ mouse sera to cryosections of WT murine palate skin; *n* = 10. (**B**) Number of mice with positive fluorescence pattern of autoantibodies for nuclear/perinuclear or basement membrane/basal keratinocytes reaction pattern on WT murine palate skin; *n* = 9–10. (**C**) Scaling of detected autoantibody fluorescence intensity observed in the sera-treated skin slides; *n* = 9–10. Scoring of fluorescence intensity was determined as 0 = negative, 1 = weakly positive (+), 2 = positive (++), and 3 = strongly positive (+++). Median was taken from 4 independent evaluations. Data are shown as mean ± SEM. Significance was determined by 2-tailed unpaired *t* test. *P < 0.05. Magnification, 20×.

To further characterize the multiorgan and systemic inflammation observed in the absence of CD18 expression on Treg, we examined serum collected from CD18^Foxp3^ mice for the presence of autoantibodies. Serum from these mice was observed to be reactive against different sites in mouse skin tissue sections (Figure 3A). Both antinuclear antibodies (ANA) — which are associated with systemic inflammatory autoimmune diseases such as lupus (28, 29), scleroderma (30), dermatomyositis (31), and Sjögren′s disease (32) — as well as autoantibodies against basal keratinocytes or basement membrane structures, which occur in autoimmune blistering skin diseases such as pemphigus (33) and bullous pemphigoid (34), were found to be present in sera from CD18^Foxp3^ mice. Autoantibodies were most commonly of the IgG type in CD18^Foxp3^ mice and were rarely observed in WT mice. Four of 10 sera contained readily detectable antinuclear autoantibodies, 5 of 10 sera were reactive with the cutaneous basement membrane and keratinocytes, and 2 out of these mice produced both types of autoantibodies. The mean autoantibody fluorescence intensity was significantly higher in CD18^Foxp3^ mice compared with littermate CD18^wt^ mice (Figure 3, B and C).

### Multiorgan inflammation in the absence of CD18 on Treg is characterized by increased T cell activation and granulocytosis.

**Figure 4:**
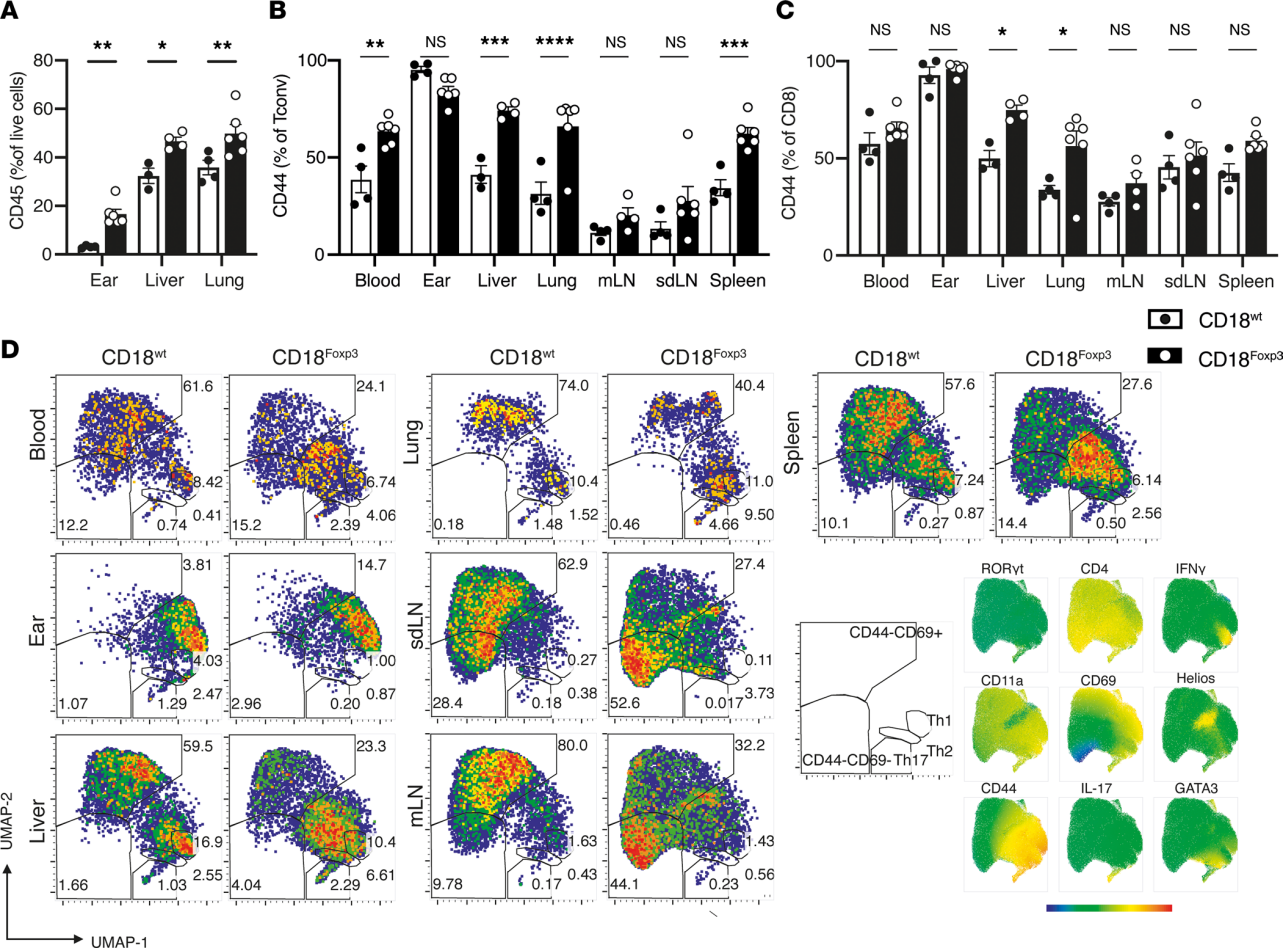
Multiorgan inflammation in CD18^Foxp3^ mice is characterized by increased immune infiltration and T cell activation. (**A**) Frequency of hematopoietic CD45^+^ cells from total live cells in ears, liver, and lungs of 12- to 13-week-old mice displaying signs of skin inflammation and littermate controls determined by flow cytometry. (**B** and **C**) Quantification of CD44 expression on Tconv and CD8^+^ T cells in multiple organs from CD18^Foxp3^ mice and littermate controls; *n* = 3–6. (**D**) UMAP of Tconv using flow cytometry data from multiple organs of CD18^Foxp3^ and CD18^wt^ mice. (**A**–**C**) Dots indicate individual mice. Data are shown as mean ± SEM, representative of at least 2 independent experiments per organ. Significance was determined by 2-way ANOVA with Šídák’s multiple-comparison test. *P < 0.05; **P < 0.01; ***P < 0.001; ****P ≤ 0.0001.

To further dissect the nature of the inflammation observed in affected CD18^Foxp3^ mice on a cellular level, we performed flow cytometric analysis of immune infiltrates in nonlymphoid organs. Analysis of the ears, livers, and lungs of mice with clinical signs of skin inflammation showed an increase in the amount of infiltrating hematopoietic cells in CD18^Foxp3^ mice compared with littermate controls (Figure 4A).

T cells play important roles in the development of autoimmunity and inflammation. Examination of T cell and B cell frequencies in lymphoid organs showed similar frequencies between genotypes (Supplemental Figure 5, A and B). Likewise, Tconv frequencies in lymphoid and nonlymphoid organs in most tissues tested were roughly equivalent between WT and CD18^Foxp3^ mice (Supplemental Figure 5C). Despite similar abundances of Tconv between genotypes, more Tconv from mice lacking CD18 expression in Treg were of an activated CD44^+^ phenotype (Figure 4B and Supplemental Figure 5D). Increases in CD8^+^ T cell activation were also observed across multiple organs (Figure 4C); however, there was no increase in the frequency of these cells producing IFN-γ or IL-17 (Supplemental Figure 5, E and F).

Multidimensional flow cytometry of lung, liver, and tissues demonstrated alterations in the composition of the Tconv population, with multiple organs showing skewing away from a Th1 response toward a Th2/Th17 based response (Figure 4D).

These increases in T cell activation and changes in Tconv compartment composition were observed across multiple sites, including the blood, liver, lungs, and spleen of mice, indicating a systemic, multiorgan immune activation and suggesting a decrease in the regulation of Tconv activation by CD18-deficient Treg.

### Deficiency of β_2_-integrins does not impair Treg migration into tissues.

β_2_-Integrins have a well-characterized and important role in the adhesion and extravasation of leukocytes into inflamed tissues (35). As such, patients with LAD-1 display persistent leukocytosis, as cells that are unable to migrate into tissue sites are trapped in circulation and lymphatic organs (36, 37). T cells lacking their functional β_2_-integrin LFA-1 have been described as having defects in their emigration into skin and other tissue sites in several disease models (38–40). To address whether dysfunctional Treg migration to tissue sites could explain the induction of the observed multiorgan inflammation and increase in Tconv activation status, we first examined Treg infiltration into the inflamed ears of CD18^Foxp3^ mice.

**Figure 5:**
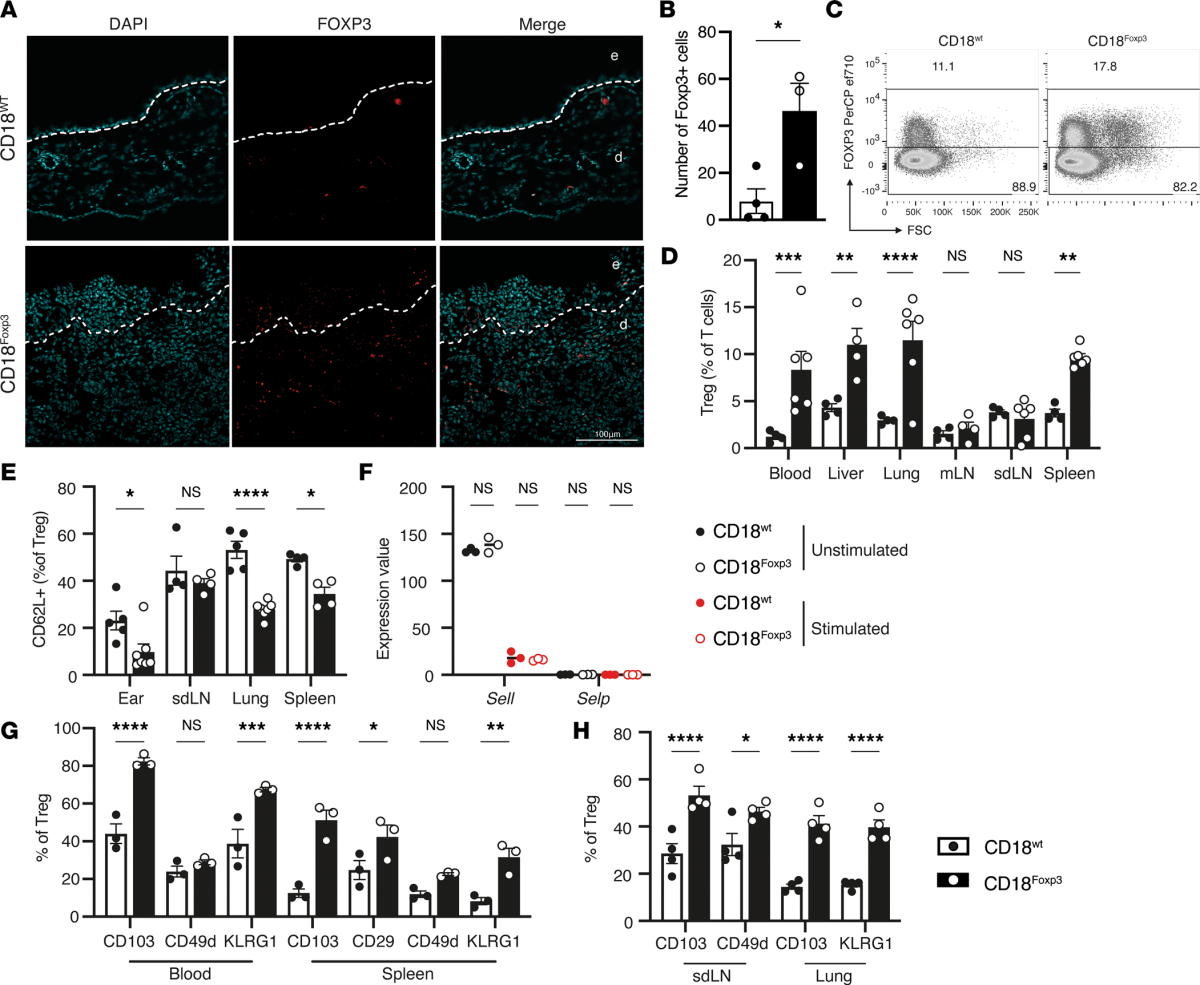
Treg-specific deletion of CD18 does not impair tissue homing of cells. (**A**) Immunofluorescence images of Treg infiltration in ears of 17- to 20-week-old mice showing nuclei marked with DAPI and Treg identified by FOXP3 expression. Images representative of 3 mice per group. Magnification, 40×. (**B**) Quantification of total number of FOXP3^+^ Treg per field of view from immunofluorescence imaging of ears of WT and CD18^Foxp3^ mice; *n* = 3–4. (**C** and **D**) Representative flow cytometry plots of FOXP3 expression on splenic CD4 T cells, and quantification of Treg frequencies as a percentage of total CD3^+^ T cells in various organs of 12- to 13-week-old mice; *n* = 3–6. (**E**) Quantification of surface expression of CD62L on FOXP3^+^ Treg from multiple organs measured by flow cytometry; *n* = 4–8. (**F**) Expression of *Sell* and *Selp* transcripts by unstimulated and stimulated CD18-sufficient and -deficient Treg; *n* = 3. (**G** and **H**) Surface expression of proteins involved in cell adhesion on CD18^wt^ and CD18^Foxp3^ Treg from blood, spleen, skin draining lymph node (sdLN), and lungs of mice; *n* = 3–4. (**B**, **D**, **E**, **G**, and **H**) Dots represent individual mice. Data are shown as mean ± SEM, representative of at least 2 independent experiments. Significance was determined by 2-tailed unpaired *t* test (**B**) or 2-way ANOVA with Šídák’s multiple-comparison test (**D**–**H**). *P < 0.05; **P < 0.01; ***P < 0.001; ****P ≤ 0.0001. e, epidermis; d, dermis.

Surprisingly, immunofluorescence imaging of tissue sections demonstrated clear infiltration of Treg into the tissue, even in the absence of Treg-expressed CD18 (Figure 5A). Quantification of infiltrating Treg revealed an increase in the number of Treg in the inflamed ears of CD18^Foxp3^ mice compared with uninflamed CD18^wt^ controls (Figure 5B). To further assess the ability of Treg to migrate in the absence of CD18, we examined the distribution of CD18^Foxp3^ Treg in various other sites by flow cytometry. Unlike the drastically reduced Treg numbers that would be expected from dysfunctional Treg migration, we observed that Treg abundance in several diverse organs was not impaired in CD18^Foxp3^ mice (Figure 5, C and D). Indeed, in contrast to WT littermates, Treg frequencies in the lung and spleen were found to be significantly increased, implicating a functional rather than a migration-specific defect of CD18-deficient Treg (Figure 5D).

We next investigated if Treg compensated for the loss of LFA-1 by upregulation of other molecules involved in adhesion and transmigration. Surface expression of L-selectin (CD62L), involved in the retention of T cells in the lymph nodes and in migration into nonlymphoid tissues (41, 42) was decreased in Treg from the ears, lungs, and spleens of CD18^Foxp3^ mice (Figure 5E). P-selectin expression was not detected in Treg (Figure 5F). Analysis of other adhesion molecules on the surface of Treg showed increased expression of surface CD29 (β_1_-integrin) on splenic Treg and CD103 on Treg from blood, spleen, lymph nodes, and lungs of CD18^Foxp3^ mice (Figure 5, G and H). KLRG1 expression was likewise increased on Treg from the blood, spleens, and lungs of CD18^Foxp3^ mice, where CD49d (VLA4) was only significantly upregulated in Treg from skin draining lymph nodes (Figure 5, G and H).

**Figure 6:**
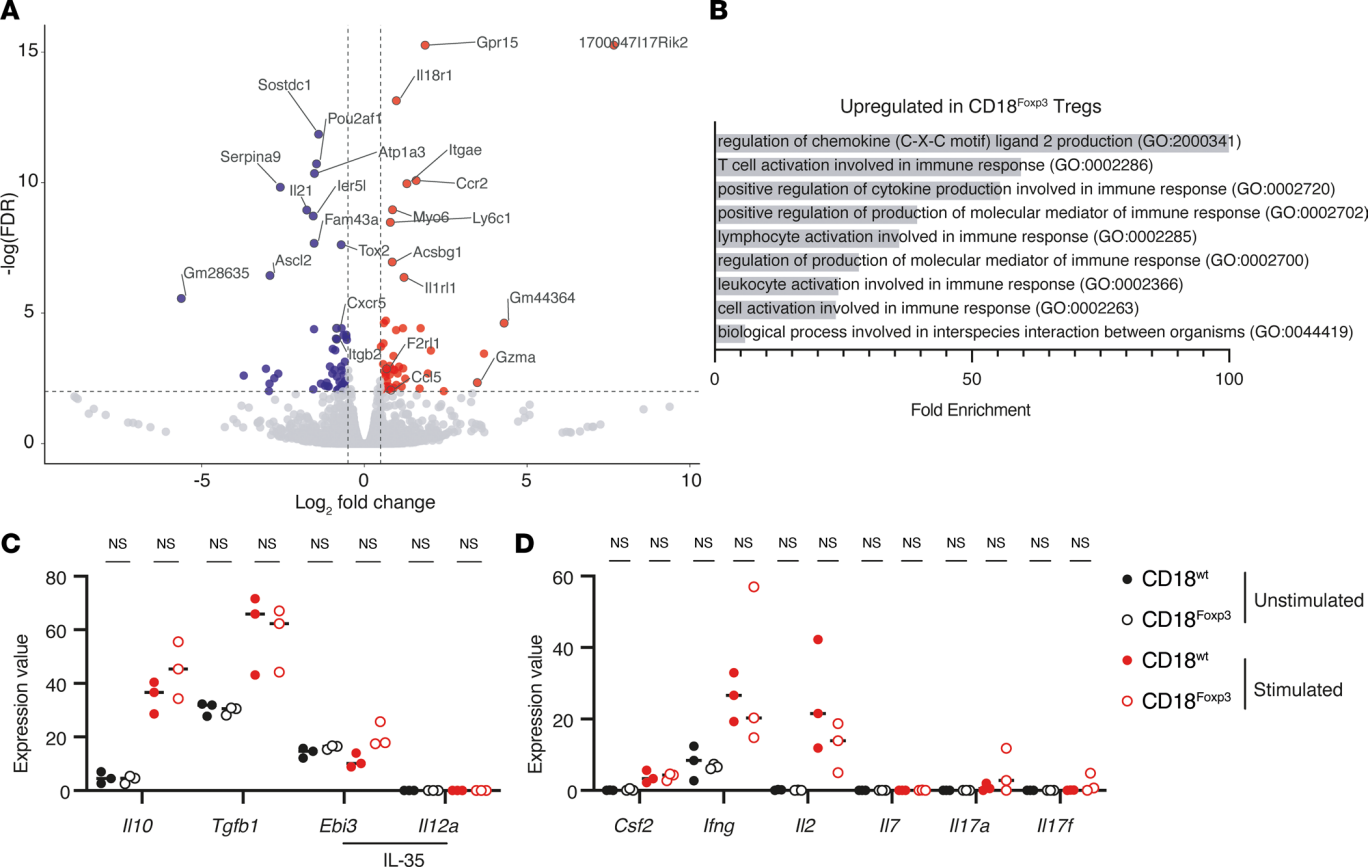
Treg lacking CD18 show altered gene expression profiles. (**A**) Volcano plot of differential gene expression between CD18^Foxp3^ and CD18^wt^ mice created using VolcaNoseR (77). Red and blue dots denote significantly up- and downregulated genes (fold change ≥ |1.5|, –log[FDR] ≥ 2 [indicated by gray lines]); *n* = 3. (**B**) Fold enrichment of the significantly enriched biological process GO terms from the genes upregulated (fold change ≥ 1.5, difference ≥ 4) in CD18^Foxp3^ mice. (**C** and **D**) Expression of Treg-associated and proinflammatory cytokine transcripts by unstimulated and stimulated CD18-sufficient and -deficient Treg; *n* = 3. Dots represent individual mice, and lines indicate the mean. Significance was determined by Fisher’s exact test with FDR correction (**A**) or 2-way ANOVA with Šídák’s multiple-comparison test (**C** and **D**).

To analyze the functional status of Treg, we performed bulk RNA-Seq on Treg isolated from CD18^Foxp3^ mice and littermate controls. Analysis of significantly differentially expressed genes (fold change ≥ 1.5, –log[FDR] ≥ 2) highlighted a number of genes where expression was altered in the absence of CD18 on Treg (Figure 6A), indicative of an altered functional state of these cells. Gene ontology (GO) analysis of the significantly upregulated genes (excluding lowly expressed genes with differential expression ≤ 4) in CD18^Foxp3^ Treg, revealed multiple enriched gene clusters, including a number of GO terms related to immune activation (Figure 6B). The most notable of these is a 100-fold upregulation of CXCL2-related genes, which are chemotactic for polymorphonuclear leukocytes and regulate lymphoid–myeloid cell crosstalk.

Given the altered gene expression profile observed when Treg lack CD18, we next aimed to determine whether defects in production of antiinflammatory cytokines by CD18-deficient Treg could also be driving the inflammation observed in these mice. We therefore analyzed the expression of these genes in both ex vivo isolated Treg in the presence and absence of additional stimulation. While the expression of *Il10* and *Tgfb1* genes was increased after stimulation as expected, there were no differences observed when Treg lacked CD18 (Figure 6C). Similarly, no differences in the expression of genes encoding for IL-35 (*Ebi3* and *IL12a*) were observed (Figure 6C). Treg production of IL-10 protein was also unaltered ex vivo (Supplemental Figure 6). The expression of transcripts encoding cytokines involved in induction of inflammation by T cells was also unaltered in the absence of CD18 Treg both with and without additional stimulation (Figure 6D). This indicates that expression of antiinflammatory cytokines is intact in the absence of CD18 on Treg.

### Treg interactions with DCs are impaired in the absence of CD18 Treg leading to the increased activation status of DC in vivo.

**Figure 7:**
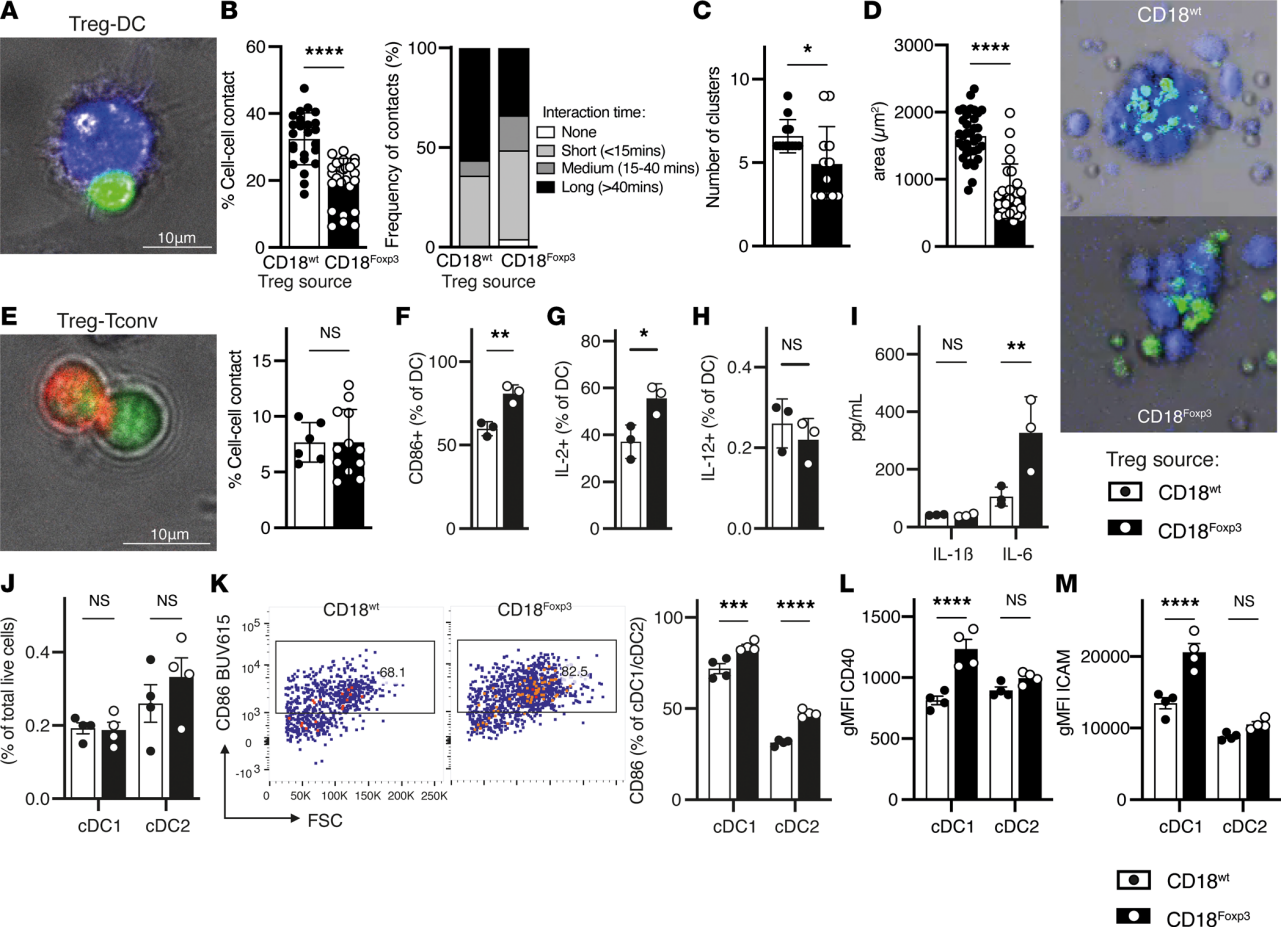
CD18-deficient Treg have dysfunctional DC interactions leading to increased DC activation in vivo. (**A**) Frequency of cell-to-cell contacts between CD18^Foxp3^ or CD18^wt^ Treg (green) and WT BMDC (blue) assessed by time-lapse microscopy for 2 hours. Individual Treg of 4 different picture sections were selected, and cell contacts with BMDC were counted with ImageJ analysis software. Total number of Treg in each section was related to the number of contacts; *n* = 2. (**B**) Interaction time of CD18-deficient Treg with BMDC. Ten different picture sections were counted every 6 minutes up to 60 minutes to determine length of contacts. (**C** and **D**) Quantification of the number and average size of DC-Treg aggregates/clusters after 24 hours of culture, measured using ImageJ analysis software. Two experiments were pooled; *n* = 2. Representative pictures of cluster formation between Treg (green) and DC ( blue) cocultures of CD18^wt^ and CD18^Foxp3^ mice. Magnification, 10×. (**E**) Representative experiment of cell-to-cell contacts between CD18^Foxp3^ or CD18^wt^ Treg (green) and WT conventional T cells (red) assessed by time-lapse microscopy. Percentage of contacts related to total cell number was determined as described in **A**; *n* = 2. (**F**–**H**) Flow cytometric quantification of DC expression of CD86, IL-2, and IL-12 in coculture with Treg; *n* = 3. (**I**) Secreted IL-1β and IL-6 measured by cytometric bead array in supernatant of DC-Treg cocultures; *n* = 3. (**J**–**M**) Quantification of flow cytometry data showing total frequencies of cDC1 and cDC2 and activation status by proportion of CD86^+^ cells and geometric mean of the fluorescence intensity (gMFI) of CD40 (**L**) and ICAM (**M**) in control and CD18^Foxp3^ mice; *n* = 4. Dots represent individual mice. Data are shown as mean ± SD (**A**–**I**) or SEM (**J**–**M**). Significance was determined by 2-tailed unpaired *t* test (**A**–**H**) or 2-way ANOVA with Šídák’s multiple-comparison test (**I**–**M**). *P < 0.05; **P < 0.01; ***P < 0.001; ****P ≤ 0.0001.

To determine whether LFA-1 deficiency specific to Treg results in impaired cell contacts with DC, we first cocultured CD18-deficient and WT Treg with syngenic BM-derived DC (BMDC) and observed cell interactions by time-lapse microscopy. The number of cell-to-cell interactions between Treg lacking CD18 and BMDC were substantially reduced compared with WT Treg-BMDC (Figure 7, A and B). Furthermore, the duration of interactions between Treg and BMDC was significantly decreased in the absence of CD18 expression on Treg, with CD18^Foxp3^ Treg forming fewer medium and long interactions with BMDC (Figure 7B). This also led to the formation of fewer Treg-DC aggregates in the absence of Treg-expressed CD18 (Figure 7C). The size of the clusters formed was also significantly reduced when cocultured Treg lacked expression of CD18 (Figure 7D). Interestingly, Treg-Tconv interactions were not altered in the absence of Treg-expressed CD18, since Treg from CD18^Foxp3^ mice formed equal numbers of contacts with Tconv (Figure 7E).

Analysis of DC after coculture with Treg revealed increased surface expression of costimulatory molecule CD86 when Treg lacked CD18 (Figure 7F). Similarly, DC expression of cytokine IL-2, which acts to promote T cell activation and expansion by DC (43), was increased in DC cocultures with CD18-deficient Treg compared with WT Treg (Figure 7G). DC expression of IL-12, involved in the polarization of a type I immune response, was unaffected by the expression of CD18 on cocultured Treg (Figure 7H). Secretion of the pleiotropic cytokine IL-6 was also increased when Treg in cocultures lacked expression of CD18, while no differences in IL-1β secretion were observed (Figure 7I). These alterations are indicative of increased DC activation after coculture with CD18-deficient Treg.

Given that Treg-Tconv interactions in vitro appeared normal and that Treg-DC interactions are known to be important in maintaining immune homeostasis (9), we wondered whether the reduced contacts between CD18-deficient Treg and DC might influence the DC activation status in vivo. Examination of splenic DC in CD18^Foxp3^ mice showed that, while the abundance of both conventional DC populations (XCR1^+^ cDC1 and SIRPα^+^ cDC2) were unaltered (Figure 7J), both cDC1 and cDC2 had increased expression of the activation marker/costimulatory molecule CD86 (Figure 7K). Interestingly, the cDC1 population from CD18^Foxp3^ also expressed significantly more CD40 and ICAM, indicative of an increased activation status (Figure 7, L and M, and Supplemental Figure 7).

## Discussion

Human LAD-1 syndrome is a rare disease attributed to mutations in the CD18 gene. These mutations lead to the deficiency of β_2_-integrin expression and impairment of leukocyte migration out of blood vessels and into tissues. As β_2_-integrin expression in some LAD1 patients differs vastly between cells, the clinical phenotype is heterogeneous and can affect almost all immune cells. Patients can suffer from a number of symptoms, including recurrent bacterial infections of the skin, lung, and mucous membranes, as well as severe periodontitis and poor wound healing. Interestingly, in addition to these immunodeficiency symptoms, patients with LAD-1 were also reported to suffer from inflammatory bowel disease, autoimmune nephritis, type 1 diabetes, and other autoimmune phenomena (44, 45). Thus, the clinical symptoms of LAD-1 combine aspects of immunodeficiency as well as autoimmunity. Until now, the only curative therapy is allogeneic hematopoietic stem cell transplantation (46, 47). Various mouse models with a constitutive KO of either an α or β subunit have been established in order to study the pathophysiology of β_2_-integrin mutations (16–21). As in patients with LAD-1, β_2_-integrins in these models are globally defective in all leukocytes, and these mice also exhibit enhanced susceptibility to infections and delayed wound healing, as well as lymphatic hyperplasia, generalized leukocyte activation, and symptoms of autoimmunity (15, 20, 21, 38). Since all leukocytes are affected in mice with a global defect in β_2_-integrins, it is difficult to dissect the cellular mechanisms of the apparently paradoxical coexistence of immunodeficiency and autoimmunity. Thus, it is of interest to generate mouse models with cell-specific deletions of β_2_-integrins (3).

In this study, we generated a Treg-specific β_2_-integrin KO mouse model. These mice suffer from spontaneous skin inflammation and hyperplasia of lymphoid organs. Since the α and β subunit pair intracellularly, a KO of CD18 results in a decreased expression of the α subunit, as well (here CD11a) (48). Skin sections of CD18^Foxp3^ mice show thickening of the epidermis according to cell infiltrations and inflammation, which indicates an important role of LFA-1 on Treg in this model. We demonstrate here that CD18-deficient Treg of CD18^Foxp3^ mice are still able to home into tissue; indeed, we found an increased presence of Treg in multiple organs. In contrast, CD11a^–/–^ mice reveal less homing, but not a complete absence of CD4^+^ cells, to secondary lymphatic tissue and decreased adhesion to high endothelial venules for leukocytes in general (49). Reduced Treg frequencies in CD11a^–/–^ mice were also observed in the inflamed nervous system in a model for EAE. In general, lymphocyte arrest and transmigration along the endothelium is exclusively mediated by LFA-1 and ICAM/JAM interactions. Rolling of neutrophils and lymphocytes along vascular endothelia and their transmigration into tissues is greatly impaired in patients with LAD-1 and in CD18^–/–^ mice, leading to blood neutrophilia and lymphatic hyperplasia (21, 39, 50, 51). In contrast, our data suggest that LFA-1 may not be required for Treg transmigration, as previously assumed (52); this loss of LFA-1 expression may also be partially compensated for by the upregulation of other integrins such as β_1_-integrins, CD103, and VLA-4, as well as other migration associated molecules, including KLRG1. However, we cannot completely rule out that cre-induced deletion of β_2_-integrins from the surface of Treg occurs only after their migration from blood into peripheral tissues. Nevertheless, the inflammatory phenotype is not due to an absence of Treg in peripheral tissues of CD18^Foxp3^ mice.

In contrast to the established role of CD18-deficient neutrophils (53, 54), much less is known about the pathogenetic relevance of Treg in patients with LAD-1. Patients with reversion mutations leading to cytotoxic T cells expressing CD18 showed increased frequencies and diminished suppressive function of LFA-1–deficient Treg, comparable with our observations in CD18^Foxp3^ mice (14). Our data indicate an upregulation of genes driving positive regulation of cytokine production in Treg, as well as increased T cell activation and immune cell infiltrates affecting multiple organs, supporting an impaired suppressive function of CD18^Foxp3^ Treg. The lack of Treg suppression leading to an altered T cell immune response has been postulated to be the driver of autoimmune bowel disease in these patients (14, 45). Lack of Treg-suppressive function in CD18^–/–^ mice was also observed by Marski et al. (55). Our use of a conditional KO confirms that this functional change in Treg is a direct result of the lack of Treg-expressed CD18, rather than a result of global immune changes due to loss of CD18 expression in multiple cell types as observed in CD18^–/–^ mice and LAD-1 syndrome. The autoimmune phenotype in CD18^Foxp3^ mice is underlined by the presence of autoantibodies. We observed increased nuclear/perinuclear autoantibody patterns, as well as the production of autoantibodies against basement membrane components and/or basal keratinocytes. The latter is found in blistering autoimmune skin diseases (56), whereas antinuclear autoantibodies are a hallmark of systemic lupus (57). Increased autoantibody production has also been observed in CD18^–/–^ mice (55). In this respect, the spontaneous phenotype of CD18^Foxp3^ mice shares many aspects with that of scurfy mice that have a mutation in the *Foxp3* locus and completely lack functional Treg (27, 58, 59). Similar to these mice, CD18^Foxp3^ mice also spontaneously develop autoantibodies and autoinflammation in multiple organs, albeit later in life and with less intensity. This, in combination with our data, suggests that the cell-intrinsically altered function of CD18-deficient Treg contributes to the multiorgan autoinflammatory phenotype in CD18^Foxp3^ mice.

Loss of other molecules involved in tissue adhesion and transmigration of T cells such as PSGL1 have also been shown to lead to the spontaneous development of autoimmunity with similar characteristics. PSGL1-deficient animals develop autoantibodies and skin fibrosis and display increased CD4^+^ T cell and DC activation (60) similar to CD18^Foxp3^ mice. However, these mice develop a much more severe disease, with increased mortality, alterations to skin vasculature, and loss of kidney function. These differences might be due to the lack of PSGL1 expression on multiple cell types such as neutrophils, macrophages, and B cells, which all use PSGL1/P-selectin interactions to promote endothelial binding and transmigration (61, 62). Since PSGL1 has also been described as a negative regulator of T cell activation (62), the lack of PSGL1 in this model may lead to an intrinsic increase in T cell activation, where increases in Tconv cell activation in CD18^Foxp3^ mice are due to extrinsic factors.

Treg lacking CD18 appear to have intact tissue migration but have altered transcriptional profiles, suggesting LFA-1–dependent intrinsic signaling defects in CD18^Foxp3^ mice. Indeed, 6 of 9 of the top enriched GO terms relate to immune activation, rather than immune suppression, as would be expected from functional Treg, implying a defect in the regulatory capacity of these cells. One of the most prominent alterations in gene expression is a significant upregulation of CXCL2-related genes that chemoattract polymorphonuclear leukocytes and other myeloid cell types (63). Since a regulation of neutrophil chemotaxis and function by normal Treg has been described before (64, 65), a function of LFA-1 on Treg may be to control myeloid cell influx into tissues. Alongside the role of LFA-1 as a prominent adhesion receptor facilitating T cell homing to sites of peripheral tissue and inflammation, LFA-1 also mediates strong adhesion to APCs by reorganizing distinct proteins within the IS (66).

In addition to leukocyte migration, β_2_-integrins have a central role in mediating T cell–APC synapse formation and T cell activation (67). Loss of LFA-1 on Tconv results in reduced contacts with DC, as previously described (68). The modulation of the activation status of APC through formation of strong and prolonged contact is a key way in which Treg exert their control of the immune response (69, 70). We observed fewer cell-to-cell contacts, as well as shorter interaction times of CD18-deficient Treg with DC in vitro. These results support previous descriptions of impaired binding between LFA-1–deficient Tconv and DC (68). It is known that LFA-1 on Treg is required for strong adhesion to DC (69, 71), which is an important mechanism by which Treg mediate immune homeostasis. This binding results in the suppression of DC priming of Tconv through multiple mechanisms, including transendocytosis of costimulatory molecules necessary for Tconv activation (72), prevention of Tconv binding to DC (69, 70, 73), and cytoskeleton rearrangement (70). Both the transendocytosis of costimulatory molecules and the formation of DC-Treg clusters that act to reduce Tconv cell binding to DC were disrupted when Treg lacked CD18 expression. This, alongside increases in DC cytokine production, is indicative of a reduced ability of CD18-deficient Treg to effectively control DC activity and, thus, the increased ability of these cocultured DC to activate both T cells and other cell types. Our in vitro observations indicate that the observed increase in DC activation in vivo in CD18^Foxp3^ mice is likely due to the inability of CD18-deficient Treg to homeostatically suppress DC activation. We hypothesize that the resulting hyperactivation of DC might contribute to spontaneous T cell activation and autoimmunity in vivo. Whether an altered suppressive function of LFA-1–deficient Treg in combination with reduced cell contacts to APC leads to the breakdown of tolerance in these mice has to be elucidated. Nevertheless, a defective interaction of Tconv with CD18^–/–^ Treg appears unlikely, since dynamic interactions between CD18-deficient Treg and Tconv were not impaired. Thus, the inability of CD18-deficient Treg to engage Tconv via LFA-1/ICAM interactions might be substituted by the unimpaired capacity of Tconv to engage ICAMs on CD18-deficient Treg via their intact LFA-1 molecules.

In conclusion, we demonstrate that β_2_-integrins are not required for Treg homing into tissue. Moreover, β_2_-integrins on Treg are important to form durable cell-to-cell contacts with APC. Reduced cell-to-cell contacts may lead to a defect in immune homeostasis in CD18^Foxp3^ mice, contributing to the increased activation of Tconv and DC. This Treg-restricted, β_2_-integrin–dependent breakdown in immune tolerance leads to spontaneous multiorgan inflammation in these mice. CD18^Foxp3^ mice demonstrate the first murine model to our knowledge to clearly identify the role of LFA-1 on Treg without interfering with other CD18-deficient immune cells in vivo and implicate Treg dysfunction as a major driver of autoimmunity observed in LAD-1 syndrome.

## Methods

### Animal models.

Mice were maintained and bred in the Central Animal Facility of the Johannes-Gutenberg-University Mainz (Mainz, Germany) under specific pathogen–free conditions using institutionally approved protocols (permission was obtained from the Landesuntersuchungsamt Koblenz). All experiments were performed with age- and sex-matched mice. CD18^wt/wt^ Foxp3^Cre^ littermates are defined as WT controls.

Mice with a Treg-specific CD18 deletion were generated as follows. CD18^fl/fl^ mice were generated with a linearized vector based on plasmid BO44.2 with a targeting construct of LoxP-FRT-neo-FRT-loxP cassette (PolyGene Transgenetics). This construct is flanked by a 5′ short and a 3′ long arm of homology containing the sequence of CD18 Exon 3. Embryonic JM8 stem cells derived from male agouti C57BL/6N blastocysts (74) were electroporated with double-digested (*NotI*+*SalI*) vector DNA. *Sca1*-digested genomic DNA (WT allele: 15 kb; targeted allele: 6.2 kb due to vector-mediated introduction of *Sca1*) of expanded clones was screened for homologous integration of the targeting sequence by Southern blot including CD18 exon 1 (probe length: 569 bp; primers: sense, 5′-CAGTCCCCATCTCCACTCAG-3′, and antisense, 5′-GGCACTCTTTGAAGCACCAA-3′) and exon 7 (647 bp; sense, 5′-ACACATGACAGCTGGGAAGA-3′, and antisense, 5′-GTCACCAACAGCGAACAGTT-3′). Recombinant ES clones were injected into blastocysts that were subsequently transferred into the uterus of a recipient B6 albino female mouse.

Chimeric offspring was selected according to the agouti coat color. Male chimera were back-crossed for 1 generation to the B6 Albino background. Agouti offspring were screened by PCR (542 bp; sense, 5′-CAAGCTCTTCAGCAATATCACGGG-3′, and antisense, 5′-CCTGTCCGGTGCCCTGAATGAACT-3′) for the presence of the Neomycin (Neo) resistance gene. Neo^+^CD18^wt/fl^ chimera were crossed with Flp deleter mice (B6 background). Derived WT and Neo^–^CD18^wt/fl^ mice were differentiated by PCR (WT: 233 bp; Neo^–^CD18^wt/fl^: 487 bp; sense, 5′-GTGACACTTTACTTGCGACCA-3′, and antisense, 5′-TGCCAATAAAGAATTTCAGAGCC-3′). Heterozygous CD18^wt/fl^ mice were further bred to obtain homozygous CD18^fl/fl^ mice. Sequencing of a PCR-amplified (sense, 5′-GACCCCTAGATCTTCCCTGC-3′, and antisense, 5′-ATAGAACCACCAACCTCGCA-3′) 1.37 kb fragment of genomic DNA encompassing the targeted region confirmed sequence integrity.

CD18^fl/fl^ mice were originally bred with Foxp3^Cre^ mice (Foxp3^tm1[cre]/Saka^) (75) crossed to C57BL/6-inbred RFP reporter mice (Gt[ROSA]26Sor^tm1Hjf^) (76) resulting in heterozygotes CD18^wt/fl^ Foxp3^Cre^ offspring, crossed back to CD18^fl/fl^ background.

### Organ processing.

Spleens, lungs, skin-draining, and mesenteric lymph nodes were mashed with a syringe plunger (Braun) through a 40 μm cell strainer (EASYstrainer; Greiner Bio One) using 20 mL of buffer (PBS with 2% FCS). Single-cell suspensions were centrifuged (300*g*, 8 minutes, 4°C) and resuspended in appropriate buffer volume. Splenic and lung erythrocytes were lysed using Gey’s lysis buffer.

Blood was collected by cardiac puncture into tubes containing 0.5 mM EDTA. Cells were centrifuged (300*g*, 8 minutes, 4°C) as above and erythrocytes lysed with Gey’s lysis buffer.

Livers were mashed with a syringe plunger through a fine metal sieve. Cell suspensions were then centrifuged (400*g*, room temperature [RT]). Lymphocytes were then collected by centrifugation (870*g* at RT) through a Percoll (MilliporeSigma) bilayer (40% and 70% in DMEM; Thermo Fisher Scientific) and washed twice with buffer (PBS 2% FCS with 2 mM EDTA) before resuspension in appropriate buffer volume.

For isolation of leukocytes from ear skin, tissue was cut into small pieces and digested for 90 minutes at 37°C while shaking using 2 mg/mL Collagenase A (Roche), 20 μg/mL DNase I (Roche), and 1 mg/mL Dispase II (Roche) in RPMI with 10% FCS. Tissue was incubated for a further 10 minutes with the addition of 10 mM Na-EDTA (Roche). Samples were filtered and washed using 70 μm cell strainers before single-cell suspensions were centrifuged (300*g*, 8 minutes, 4°C) and resuspended in appropriate buffer volume.

For DC isolation from spleen, splenic tissue was cut into small pieces and digested for 30 minutes at 37°C while shaking using 800 U/mL collagenase type IV (Worthington) and 50 U/mL DNase I (Sigma-Aldrich) in RPMI 1640 (without FCS). Afterward, Na-EDTA was added to a concentration of 10 mM, and tissue was incubated further for 10 minutes. Cell suspensions were filtered via a 70 μm cell strainer until staining for flow cytometry.

### Immune cell isolation for time-lapse microscopy.

Splenic DC were isolated by negative immune-magnetic selection using the Pan Dendritic Cell Isolation Kit (Miltenyi Biotec). Splenic Treg and naive T cells were isolated using the CD4^+^CD25^+^ Treg isolation kit and naive CD4^+^ isolation kit, respectively. All cell isolations were performed as recommended by the manufacturer (Miltenyi Biotec).

### Transcriptome analysis of CD4^+^Foxp3^+^ T cells.

Splenic RFP^+^CD4^+^ T cells (each about 1 × 10^5^ per sample) were sorted by flow cytometry using a FACSAriaII (BD Biosciences) and lysed (RLT PlusLysis Buffer; Qiagen). Cells were incubated overnight with recombinant human IL-2 (5 U/mL). In parallel assays, agonistic anti-CD3 (1 μg/mL, BioLegend, catalog 100238) and anti-CD28 (2 μg/mL, BioLegend, catalog 102116) antibody were applied. Total RNA was purified using the RNeasy Plus Mini Kit as recommended (Qiagen). Isolated RNA was quantified employing a Qubit 2.0 fluorometer (Invitrogen), and RNA quality was determined using a Bioanalyzer 2100 with a RNA 6000 Pico chip (both from Agilent). Then, 10 ng of total RNA (integrity > 8) was used to generate barcoded mRNA-seq cDNA library using the NEBNext Poly(A) mRNA Magnetic Isolation Module and NEBNext Ultra II RNA Library Prep Kit for Illumina (both from NEB) with a final amplification of 15 PCR cycles. Afterward, RNA was quantified using the Qubit HS assay kit (Thermo Fisher Scientific). The library size distribution was determined using the Bioanalyzer HS DNA assay (Agilent). Then, barcoded RNA-Seq libraries were clustered using the HiSeq Rapid SR Cluster Kit v2 (Illumina) using 8 pM, and 59 bps were sequenced on the Illumina HiSeq2500 using HiSeq Rapid SBS Kit v2 (59 cycles). Primary sequencing results were processed according to the Illumina standard protocol. Afterward, sequence reads were trimmed (deleting adapter sequences) and processed using the CLC Genomics Workbench software (v20.0, default settings; Qiagen). Reads were aligned to GRCm38 genome. Differentially expressed genes with a fold change ≥ |1.5| and –log(FDR) ≥ 2 were calculated using CLC Genomics workbench. Analysis of the enrichment of GO terms within the upregulated genes was performed using the GO Resource and PANTHER Overrepresentation Test (73–75) (annotation version and release date, GO Ontology database DOI: 10.5281/zenodo.6399963 Released 2022-03-22), using Fisher’s exact test with FDR correction.

### BMDC.

Femurs and tibia of mice were taken, and bones were sterilized for 30 seconds with 70% ethanol. BM was flushed out with a 23 G needle in PBS 2% FCS (PAN Biotech), 100 U/mL penicillin/streptomycin (Pen/Strep; Invitrogen), 2 mM EDTA. BM cells (5 × 10^5^/mL) were seeded in 6-well plates (Greiner Bio-One) in IMDM culture medium (Thermo Fisher Scientific) (Supplemented with 5% FCS [PAN], 2 mM L-glutamine (Thermo Fisher Scientific), 100 U/mL penicillin, 100 μg/mL streptomycin, 50 μM β-mercaptoethanol (MilliporeSigma), and 10 ng/mL recombinant murine GM-CSF [Miltenyi Biotec]). Culture medium was replaced every 3 days of culture. On day 7, BMDC were used for experiments.

### Histology.

For H&E staining, mouse ears and tails were fixed in 4.5% formaldehyde overnight and embedded in paraffin. Rewaxed and rehydrated tissue sections (2 μm) were stained with Gill III Hematoxylin for 2.5 minutes, washed, and stained with 0.5% eosin Y for 1 minute afterward. Slides were placed in 95% ethanol 2 times and transferred into 100% ethanol for 2 minutes. Slides were incubated with xylene I/II for 2 minutes, dried, and covered with mounting medium (Consul Mount, Thermo Fisher Scientific). Pictures were taken with an Invitrogen EVOS M7000 microscope.

For immunofluorescence histology, cryosections of skin were fixed and stained with CryoFixation and CryoStainer of xZell, as recommended by the manufacturer (xZell, Singapore). To detect Treg populations, sections were incubated with PE Foxp3 antibody. DAPI was used to stain nuclei. Immunofluorescence imaging was performed with Leica Thunder 3D Tissue Imager. Pictures were analyzed with ImageJ (NIH).

### Indirect immunofluorescence.

Palate skin cryosections of C57BL/6N mice were fixed in acetone for 10 minutes, rehydrated in 1× TBS, and blocked with 5% goat serum in TBS for 30 minutes at RT. Primary antibody was incubated for 1 hour and, afterward, serum of CD18^Foxp3^ and WT littermates was given onto the skin slides for 1 hour. Slides were washed 3 times with TBS, and secondary anti–mouse IgG antibody (anti–mouse IgG antibody Dianova; catalog 115-546-008) was applied onto serum-treated slides for 1 hour (anti–mouse IgG antibody Dianova diluted 1:400 in 1× TBS) to detect autoantibodies. Slides were covered with DAKO-fluorescence mounting medium. Fluorescence pictures were taken using an Axioskop 40 (Zeiss) and the corresponding software.

### Time-lapse microscopy.

Magnetic isolated (MACS) CD4^+^CD25^+^ Treg of CD18^Foxp3^ mice and WT littermates (CFSE), conventional WT CD4^+^CD25^–^ T cells (CellMask orange), and BMDC (CellTrace violet) were stained with fluorescence dye for 10 minutes at 37°C (each 5 μM; Thermo Fisher Scientific). Treg and BMDC or Treg and Tconv were cocultured in an Ibidi 8-well plate. Cell cultures were kept under 5% CO_2_, 37°C, and 90% humidity in an OkoLabs environmental incubator (H-301K environmental chamber, Oko Touch, Oko Pump, T-Control and CO_2_ control, OkoLabs) on the microscope table. Cell interaction was monitored by CLSM using a Leica TCS SP8 and was performed with a 20× 0.75 NA objective with 405 nm and 488 nm excitation, and emission windows of 415 to 478 nm and 498 nm to 578 nm, for respective CellTrace Violet and CSFE detection; it was also performed with scanning Differential Interference Contrast transmission imaging in a 580 μm × 580 μm frame format with 400 lines per second, 1.14 μm/pixel (512 × 512 pixel per frame) and with 2 times averaging per line, with a frame acquisition of every 6 minutes per selected position within the chamber over 12 hours. Cell interactions were analyzed with ImageJ.

### Cocultures and cytometric bead array (CBA).

Splenic MACS isolated CD4^+^CD25^+^ Treg (Miltenyi Biotec, 130091041) of CD18^Foxp3^ mice and WT littermates, as well as MACS isolated DC (Miltenyi Biotec, 130100875) from WT mice were cocultured (3:1; DC/Treg) in RPMI from Thermo Fisher Scientific (supplemented with 10% FCS [PAN Biotech], 100 U/mL penicillin, 100 μg/mL streptomycin, 50 μM β-mercaptoethanol, 50 μM natrium pyruvate; Thermo Fisher Scientific) for 48 hours (5% CO_2_, 37°C). Cytokine analysis of the supernatant was performed with CBA flex set system (BD Biosciences; IL-6, 519004153; IL-1β, 51-9005788) as described. Beads were detected with an Attune flow cytometer and analyzed with FCAP Array software.

### Antibodies.

The following antibodies and dyes were used for immunofluorescence, time-lapse microscopy, and flow cytometric analysis of cells. For immunofluorescence, FOXP3 PE (BD Pharmingen, catalog 560414), DAPI (Roth, catalog 6335.1), goat-serum BIOZOL (catalog JIM-005-000-121), and secondary anti–mouse IgG antibody Dianova (catalog 115-546-008) were used. For time-lapse microscopy, CFSE (catalog C34554), CellMask orange (catalog C10045), and CellTrace violet (catalog C34557) were used (all dyes were from Thermo Fisher Scientific). For flow cytometry, CD3 BUV615 (BD Biosciences, catalog 751443), CD4 BUV737 (BD Biosciences, catalog 612844), CD8 BV650 (BioLegend, catalog 100742), CD8 BV711 (BioLegend, catalog 100748), CD11a PE-Cy7 (BioLegend, catalog 153108), CD11b BV421 (BioLegend, catalog 101251), CD11c APC (BioLegend, catalog 117309), CD11c APC-R700 (BD Biosciences, catalog 565872), CD16/23 (Fc Block, made in house), CD18 APC (BD Biosciences, catalog 562828), CD29 APC (Thermo Fisher Scientific, catalog 17-0291-80), CD40 BUV395 (BD Biosciences, catalog 565202), CD44 APC-R700 (BD Biosciences, catalog 565480), CD44 BUV737 (BD Biosciences, catalog 612799), CD45.2 BUV805 (BD Biosciences, catalog 741957), CD69 BUV395 (BD Biosciences, catalog 740220), CD86 BUV615 (BD Biosciences, catalog 751557), CD86 FITC (eBioscience, catalog 11086285), CD103 BV711 (BioLegend, catalog 121435), CD103 PE (BioLegend, catalog 121405), fixable viability dye eFluor 780 (Thermo Fisher Scientific, catalog 65-0865-18), FOXP3 PerCP-eFluor 710 (Thermo Fisher Scientific, catalog 46-5773-82), GATA3 APC (Thermo Fisher Scientific, catalog 50-9966-42), Helios FITC (BD Biosciences, catalog 563590), ICAM FITC (BioLegend, catalog 116106), IFN-γ BV605 (BioLegend, catalog 505840), IL-2 PE-Cy7 (Thermo Fisher Scientific, catalog 25-7021-82), IL-10 PE (BD Biosciences, catalog 554467), IL-12 APC (BD Biosciences, catalog 554480), IL-17 BV421(BioLegend, catalog 506926), KLRG1 PE-Dazzle (BioLegend, catalog 138424), MHCII BV786 (BD Biosciences, catalog 742894), RORγt PE (BD Biosciences, catalog 562607), Sirp1a PE-Cy7 (BioLegend, P84, catalog 144008), and XCR1 BV650 (BioLegend, catalog 148220) were used.

### Staining for flow cytometry.

Single-cell suspensions were stained with an antibody cocktail containing Fc Block and surface markers in FACS buffer (PBS 2% FCS with 2 mM EDTA) for 30 minutes at 4°C. Cells were washed with FACS buffer and fixed using the eBioscience Foxp3/Transcription Factor Staining Buffer Set (Thermo Fisher Scientific) as per the manufacturer’s instructions. Cells were then washed twice with permeabilization buffer (Thermo Fisher Scientific) and stained intracellularly for FOXP3 in permeabilization buffer for 30 minutes at 4°C. After staining, cells were washed with permeabilization buffer and resuspended in FACS buffer for analysis. Where intracellular cytokine staining was necessary, single-cell suspensions were first restimulated for 4 hours at 37°C using 50 ng/mL PMA (MilliporeSigma), 1 μg/mL ionomycin (MilliporeSigma) in the presence of monensin (Thermo Fisher Scientific) before proceeding with surface staining, fixation, and intracellular staining.

Flow cytometry data were collected using the BD FACS Symphony (BD Biosciences). Data were analyzed using FlowJo v10.8.1 (BD Biosciences).

### Experimental design and blinding.

For monitoring of signs of skin inflammation, both animal caretakers and researchers were blinded to the genotypes of animals. For all ex vivo experiments, including histological analysis, indirect immunofluorescence, and flow cytometry, samples were randomized, and researchers were likewise blinded to mouse genotype.

### Data availability.

RNA-Seq data generated for this study were deposited in the NCBI’s Gene Expression Omnibus database (GEO GSE215787).

### Statistics.

Data were analyzed using GraphPad PRISM v 9.2.0 software (GraphPad Software Inc.). For comparisons between only 2 groups, statistical significance was assessed using an unpaired, 2-tailed *t* test. For data sets where comparisons between multiple groups were necessary, statistical significance was assessed using either a 2-way ANOVA with Šídák’s multiple-comparison test, Fisher’s exact test with FDR correction, or multiple unpaired 2-tailed *t* tests corrected for multiple comparisons by the 2-stage step-up (Benjamini, Krieger, and Yekutieli) method. In all cases, significance was defined as *P* < 0.05.

### Study approval.

Mice were sacrificed for organ retrieval according to § 8 TierSchG. All animal experiments were performed after approval by the regional regulatory authorities (Landesuntersuchungsamt LUA, Rheinland-Pfalz, Koblenz, reference no. G20-1-113). The *Guide for the Care and Use of Laboratory Animals* (National Academies Press, 2011) was followed. Animal experiments were performed under the supervision of the authorized investigators in accordance with the European Union normative for care and use of experimental animals with all relevant ethical regulations.

## Author contributions

TK, ASW, TB, and SG wrote and edited the manuscript. TK, ASW, SSCH, NK, KH, JS, M Bros, EV, EH, HCP, and KM performed experiments. TK, ASW, AHE, MK, MH, EH, MH, and FB analyzed data. TK, ASW, NK, TB, and SG designed experiments. AW, M Bednarczyk, and SG constructed the mouse model. TB and SG conceptualized the study and secured funding. All authors contributed to the interpretation of the results. The order of co–first and co–senior authors who provided equal input to this manuscript was determined alphabetically.

## Supplementary Material

Supplemental data

## Version 1 11/08/2022

In-Press Preview

## Version 2 12/22/2022

Electronic publication
