# Supplementary material for: Impaired Treg-DC interactions contribute to autoimmunity in leukocyte adhesion deficiency type 1
Source: JCI Insight. 2022 Dec 22;7(24):e162580. doi: 10.1172/jci.insight.162580 (PMC9869970; doi:10.1172/jci.insight.162580)
Supplement: Supplemental data [file jciinsight-7-162580-s140.pdf]

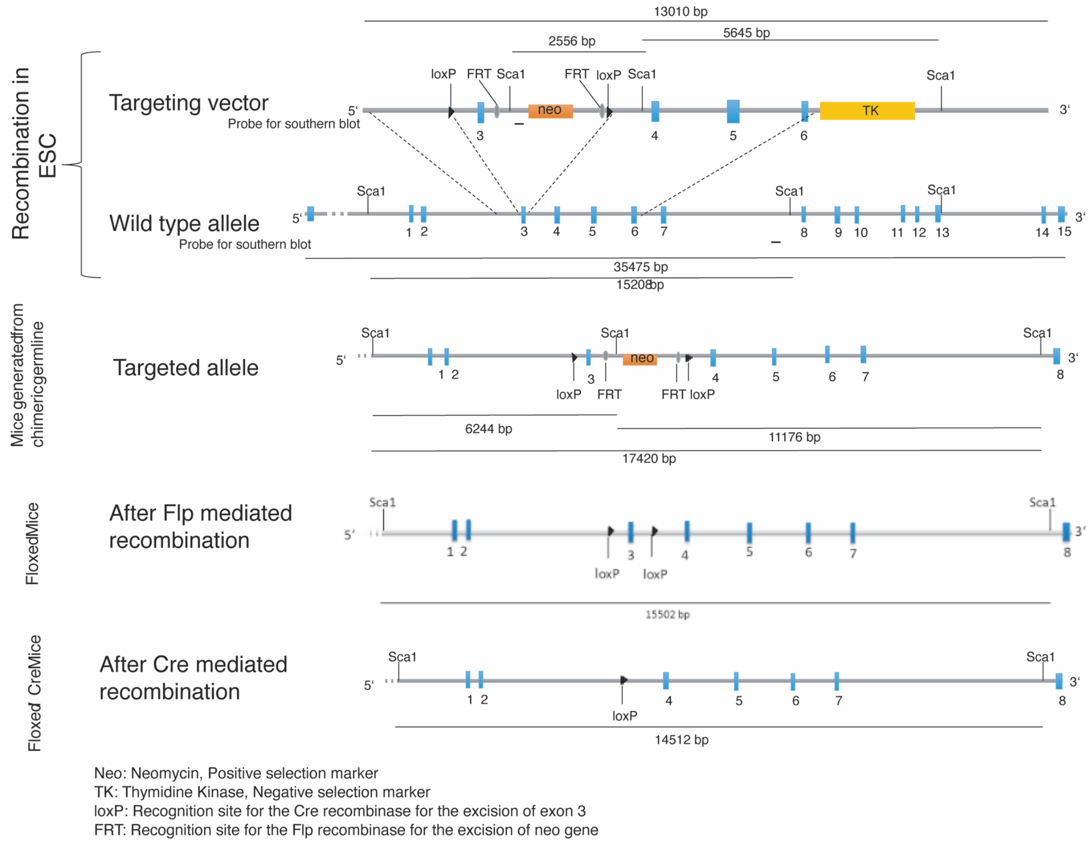

Supplementary figure 1 **Recombination strategy for the generation of cell-specific Cre-mediated CD18 knockout.** The targeting construct comprises the sequence of exon 3 of the CD18 gene containing a loxP-FRT-neo-FRT-loxP cassette and long arm of homology, which is recombined with wild type embryonic stem cell DNA. Neomycin resistance gene facilitates the selection of recombined embryonic cells. After Flp recombination the Neomycin cassette was removed and Cre mediated recombination deleted exon 3 of the CD18 gene locus, leading to complete CD18 deletion in a specific cell type.

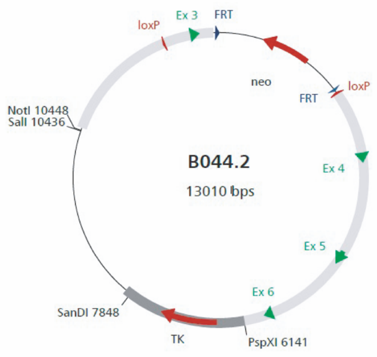

Supplementary figure 2 **BO44.2 vector construct.** The 13.010 bp long vector (Polygene) containing CD18 exon 3-6 long arm of homology. Exon 3 containing Neo-cassette with FLP sites were flanked with loxP. Not1 and Sal1 linearizing restriction sites were electroporated with the embryonic stem cells (clone JM8).

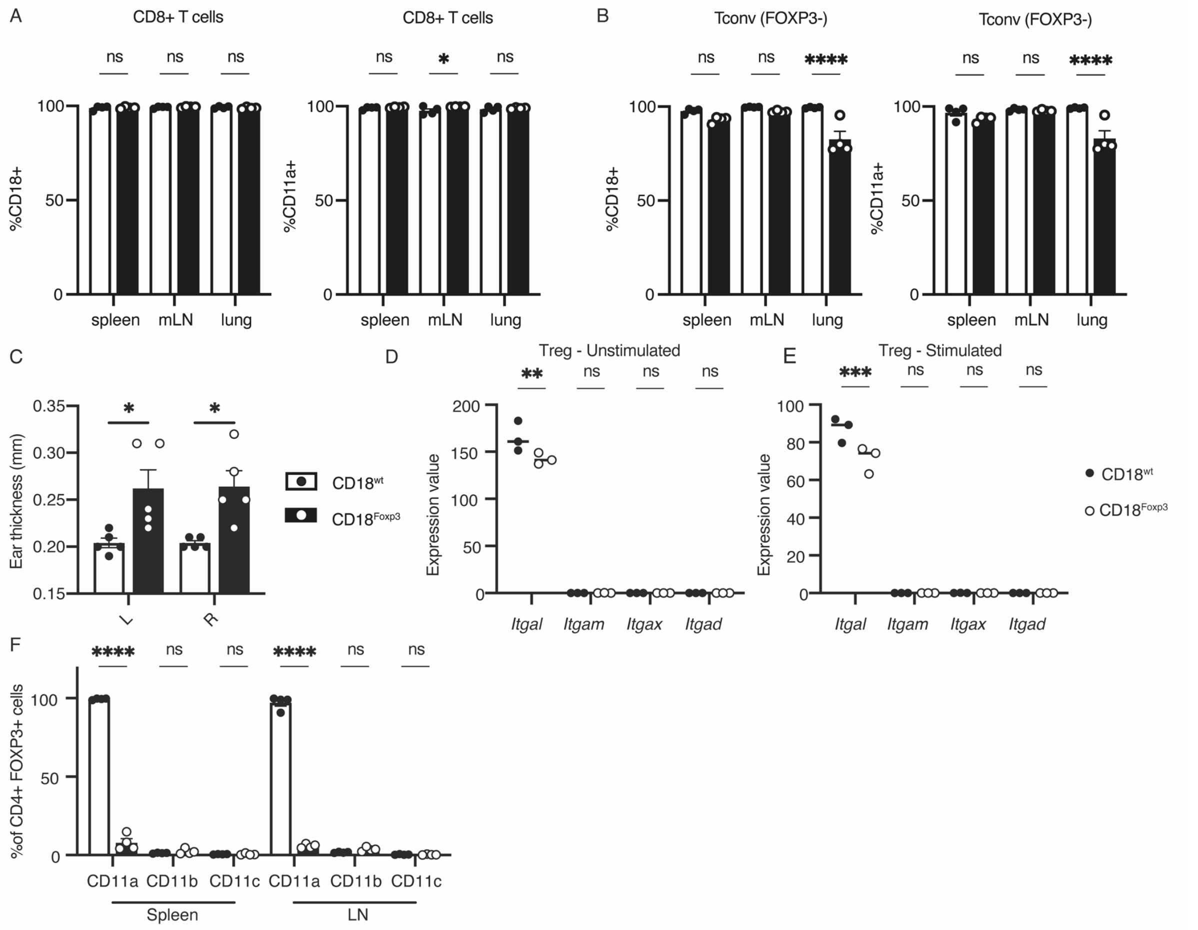

Supplementary figure 3 **CD18 expression is intact in non-Treg T cells** CD18 and CD11a expression in **(A)** CD8 T cells and **(B)** CD4 Tconv (FOXP3-) from multiple tissues measured by flow cytometry. **(C)** Ear thickness of left (L) and right (R) ears of $CD18^{wt}$ and $CD18^{Foxp3}$ mice. **(D)** Expression of transcripts of alpha integrin partners of Itgb2 in unstimulated and **(E)** stimulated Tregs isolated for $CD18^{wt}$ and $CD18^{Foxp3}$ mice, n=3. **(F)** Quantification of surface expression of alpha integrin proteins known to form heterodimers with CD18 on Tregs from spleens and lymph nodes (LN) of $CD18^{wt}$ and $CD18^{Foxp3}$ mice, n=3. Dots indicate individual mice (A-C,F) representative of at least two independent experiments. Significance was determined by 2-way ANOVA with Šídák's multiple comparisons test, ns, $p>0.05$; *, $p<0.05$; **, $p<0.01$; ****, $p\leq0.0001$.

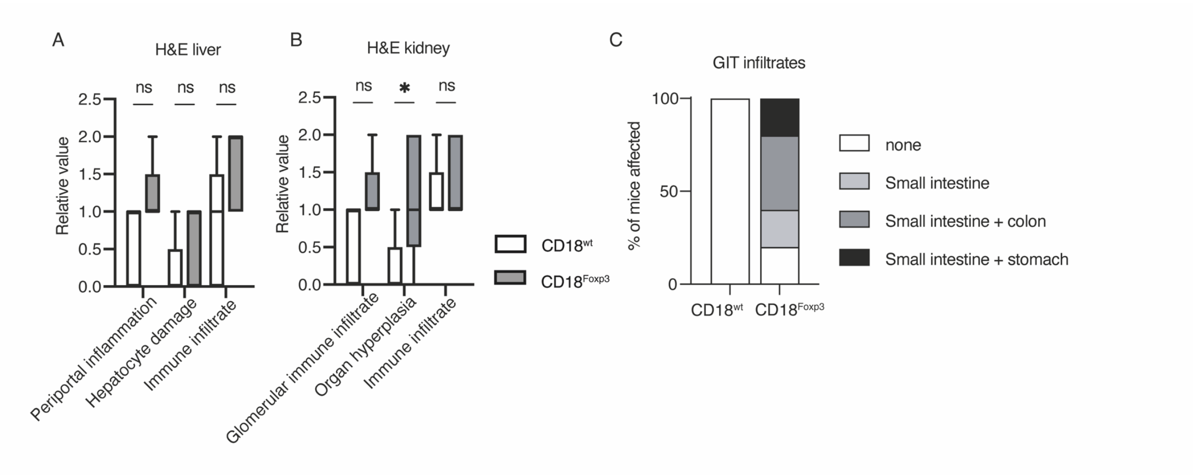

Supplementary figure 4 **CD18^{Foxp3} mice have altered immune infiltrates.** Quantification of H&E staining from **(A)** liver and **(B)** kidney samples from CD18^{wt} and CD18^{Foxp3} mice n=5. **(C)** Frequency of mice with immune infiltrates in the gastrointestinal tract (GIT) identified by H&E staining n=5. (A,B) Box plots extend from the 25th to the 75th percentile, whiskers indicate the 5th and 95th percentiles. (C) Bars indicate frequency of mice with immune infiltrates. (A,B) Significance was determined by 2-way ANOVA with Šídák's multiple comparisons test, ns, $p>0.05$; *, $p<0.05$.

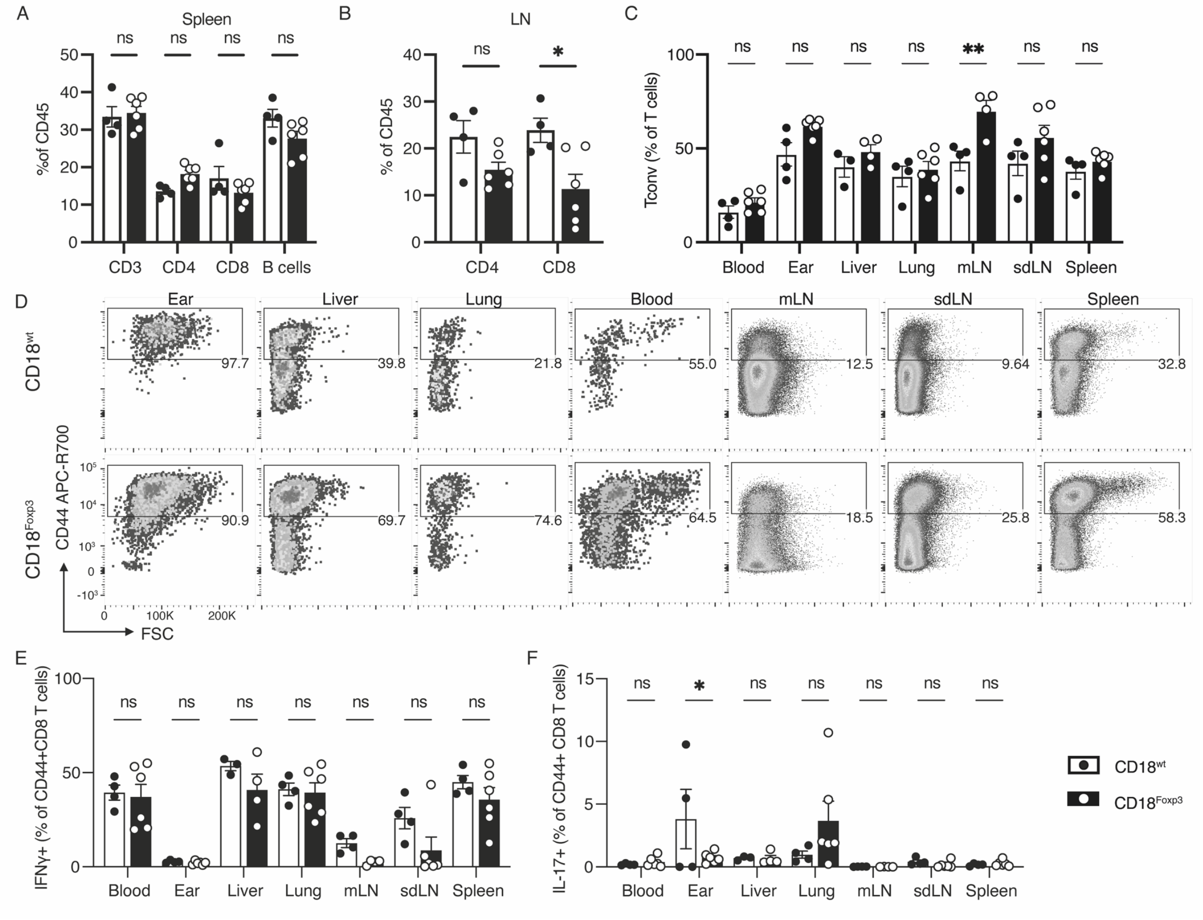

Supplementary figure 5 **CD18^{Foxp3} mice display normal T cell frequencies but increased activation.** Frequency of **(A)** splenic lymphocyte and **(B)** lymph node T cell populations measured by flow cytometry. **(C)** Conventional (FOXP3-) CD4 T cells (Tconv) in selected lymphoid and non-lymphoid organs of CD18^{Foxp3} mice.

(D) Representative flow cytometry plots showing CD44 expression of Tconv in multiple organs. **(E)** Quantification of IFNγ and **(F)** IL-17 expression by CD8 T cells from CD18^{wt} and CD18^{Foxp3} mice after ex vivo restimulation n=4-6. Dots represent individual mice, bars show the mean ± SEM. (A-C,E-F) Significance was determined by 2-way ANOVA with Šídák's multiple comparisons test, ns, p>0.05; *, p<0.05; **, p<0.01; ***, p<0.001 ****, p≤0.0001.

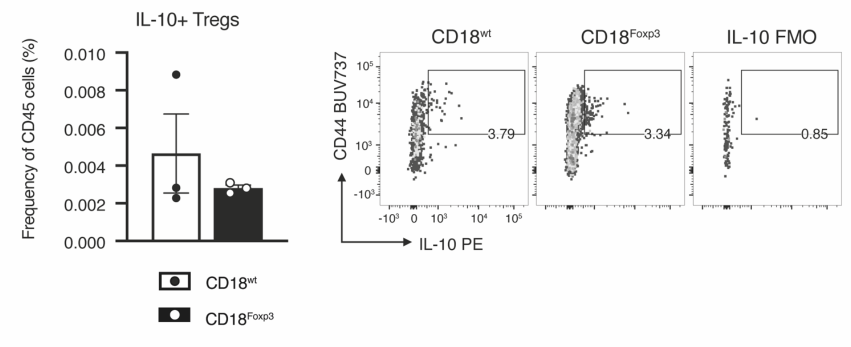

Supplementary figure 6 **IL-10 expression is not affected by the loss of CD18. (A)** Quantification and **(B)** representative flow cytometry plots showing the frequency splenic IL-10 expressing Tregs from the total CD45 cell population of $CD18^{wt}$ and $CD18^{Foxp3}$ mice. An IL-10 FMO (fluorescence minus one) was used to confirm true staining for IL-10. n=3. Dots represent individual mice, bars show the mean ± SEM. Significance was determined by two-tailed unpaired t test. ns, $p>0.05$.

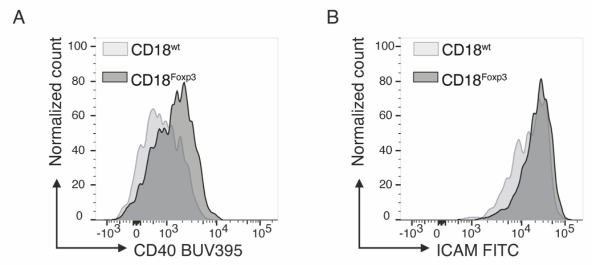

Supplementary figure 7 **Increased expression of DC activation markers ex vivo.** Representative histograms showing the fluorescence intensity of **(A)** CD40 and **(B)** ICAM on splenic cDC1 cells from CD18^{wt} (light gray) and CD18^{Foxp3} (dark gray) mice.
